# Distraction from pain: The role of selective attention and pain catastrophizing

**DOI:** 10.1002/ejp.1634

**Published:** 2020-08-13

**Authors:** Katharina M. Rischer, Ana M. González‐Roldán, Pedro Montoya, Sandra Gigl, Fernand Anton, Marian van der Meulen

**Affiliations:** ^1^ Department of Behavioural and Cognitive Sciences Research Institute of Health and Behaviour University of Luxembourg Esch‐sur‐Alzette Luxembourg; ^2^ Cognitive and Affective Neuroscience and Clinical Psychology Research Institute of Health Sciences (IUNICS) and Balearic Islands Health Research Institute (IdISBa) University of the Balearic Islands (UIB) Palma Spain

## Abstract

**Background:**

Previous research has demonstrated the efficacy of cognitive engagement in reducing concurrent pain. However, little is known about the role of individual differences in inhibitory control abilities and negative pain‐related cognitions in modulating the magnitude of this type of distraction from pain.

**Methods:**

In a pain distraction paradigm, 41 participants completed a working memory task with both a demanding high load condition (2‐back) and an easy low load condition (0‐back), while receiving warm or painful thermal stimuli to their left forearm. To control for individual differences in sensitivity to pain and perceived task difficulty, nociceptive stimulus intensity and task speed were individually calibrated. Additionally, participants completed a set of cognitive inhibition tasks (flanker, go/nogo, Stroop) and questionnaires about negative pain‐related cognitions (fear of pain, pain catastrophizing) prior to the distraction paradigm.

**Results:**

As expected, engaging in the high load condition significantly reduced perceived intensity and unpleasantness of nociceptive stimuli, compared to the low load condition. The size of the distraction effect correlated significantly with better cognitive inhibition and selective attention abilities, as measured by the flanker task. A moderation analysis revealed a significant interaction between pain catastrophizing and performance in the flanker task in predicting the distraction effect size: Participants who performed well on the flanker task showed more pain reduction, but only when they were average to high pain catastrophizers.

**Conclusions:**

Selective attention abilities and pain catastrophizing seem to be important factors in explaining individual differences in the size of the analgesic response to a distractive task.

**Significance:**

Understanding which factors influence the effectiveness of cognitive engagement in distracting from pain could help to optimize its therapeutic application in patient care. This study shows that a complex interplay of cognitive inhibition abilities, specifically selective attention, and negative pain‐related cognitions, such as pain catastrophizing, modulate the magnitude of the distraction effect.

## 1 INTRODUCTION

Distraction through cognitive engagement is a commonly used form of pain inhibition (McCaul & Malott, 1984), yet the reported magnitude of the analgesic response varies between studies (Verhoeven, Van Damme, Eccleston, Van Ryckeghem, Legrain & Crombez, 2011), with some studies finding large effects (Buhle, Stevens, Friedman, & Wager, 2012; Buhle & Wager, 2010) and other studies reporting smaller (McCaul, Monson, & Maki, 1992) or no effects (Goubert, Crombez, Eccleston, & Devulder, 2004). While this divergence in findings may partly have resulted from differences in experimental paradigms and populations, emerging evidence suggests that individual differences in executive functions, specifically cognitive inhibition abilities, shape the perception of pain (Karsdorp, Geenen, & Vlaeyen, 2014; Oosterman, Dijkerman, Kessels, & Scherder, 2010; Oosterman, Traxler, & Kunz, 2016; Zhou, Després, Pebayle, & Dufour, 2015), and may have a protective effect against pain‐induced interference on task performance (Verhoeven et al., 2011; Verhoeven, Dick, Eccleston, Goubert, & Crombez, 2014). Yet, the extent to which cognitive inhibition abilities may directly influence the efficacy of a distractive task is still unclear (Verhoeven et al., 2011, 2014). [Correction added on 26 August 2020 after first online publication: The names of the authors in “Verhoeven et al., 2011” found in the 1st paragraph and reference list were incorrect and have been updated in this version.]

Other factors that have been shown to influence the effectiveness of task‐related analgesia are negative pain‐related cognitions, such as pain catastrophizing and fear of pain, probably by promoting hypervigilance to pain (Van Damme, Crombez, Eccleston, & Roelofs, 2004). However, reports have been inconsistent with regard to the direction and magnitude of this modulation. While some studies have associated pain catastrophizing with a reduced distraction effect (Heyneman, Fremouw, Gano, Kirkland, & Heiden, 1990; Prins, Decuypere, & Van Damme, 2014; Verhoeven, Goubert, Jaaniste, Van Ryckeghem, & Crombez, 2012), other studies found either no such modulation (Van Damme, Crombez, Van De Wever, & Goubert, 2008; Van Ryckeghem, Crombez, Van Hulle, & Van Damme, 2012), reported that the effects of pain catastrophizing may diminish over time (Campbell et al., 2010) or depend on the participants’ motivation (Verhoeven et al., 2010). Schreiber et al. (2014) even reported greater distraction analgesia in higher than in lower catastrophizing chronic pain patients.

In the present study, we examined the effect of distraction on the perception of pain by delivering warm and painful heat stimuli during a working memory task with different levels of cognitive load. To control for individual differences in sensitivity to pain and perceived task difficulty, nociceptive stimulus intensity and task speed were individually calibrated. Additionally, we measured cognitive inhibition skills with a set of tasks (flanker, go/nogo, Stroop) and assessed negative pain‐related cognitions (i.e., pain catastrophizing and fear of pain) using questionnaires. We expected that individuals with better inhibition abilities would show a stronger attentional focus on the working memory task and better resist the impulse to turn their attention to the incoming nociceptive stimulus, which should be reflected by a larger distraction effect (i.e., lower intensity and unpleasantness ratings for painful stimuli when completing the high load as compared to the low load task). Based on the literature, we had no clear hypothesis regarding the influence of negative pain‐related cognitions on the distraction effect.

## 2 METHODS

### 2.1 Participants

Forty‐One students from the University of Luxembourg participated in the study. All participants had normal or corrected‐to‐normal vision, and self‐reported to be free from acute or chronic pain and any neurological or cardiovascular conditions. Other exclusion criteria included a history of repetitive fainting, and injuries or large tattoos on the volar surface of their left arm where heat stimuli were applied. Furthermore, participants were instructed not to take any pain medication or drugs (including alcohol) 48 hr prior to the experimental session. Participants were required to fluently speak either English or German; the experimental material was provided and carried out in their preferred language (39% German). Two participants were excluded from the sample (see results section), resulting in a final sample of *N* = 39 participants (19 male; age: *M* = 23.05, *SD* = 2.74; range: 18–30 years).

Participants were informed that they could win up to €5, in addition to a fixed compensation of €10, for good performance on the working memory task in order to increase their motivation (see Verhoeven et al., 2010). In reality, participants received a standard sum of €15 irrespective of their actual performance. The study was conducted in accordance with the Declaration of Helsinki. Ethical approval was obtained from the Ethics Review Panel of the University of Luxembourg.

### 2.2 Procedure

In the first part of the session, participants completed a brief demographic and health questionnaire to ensure that they met the inclusion criteria. To assess the influence of the participants’ negative pain‐related cognitions, they completed validated English or German versions of the Pain Catastrophizing Scale (PCS; Sullivan, Bishop, & Pivik, 1995) and Fear of Pain‐III Questionnaire (FPQ‐III; McNeil & Rainwater, 1998) (see below for more details). Following this, participants were seated in a sound‐attenuated room in front of a computer screen and completed three cognitive inhibition tests (see below) (all tasks are available online at cognitivefun.net). The experimenter monitored the participant and controlled the tasks from an adjacent control room with video and audio feedback.

In the second part of the session, distraction from pain was assessed in a 2 × 2 factorial within‐subject design. Participants were asked to perform an n‐back working memory task with two different cognitive loads; a high load (2‐back task) and a low load (0‐back task) condition, while they received thermal stimuli (warm or painful) to their left forearm. Prior to this, participants were given the chance to practice both conditions of the task, followed by an on‐line calibration of the presentation speed, to account for differences in task difficulty between participants. Moreover, nociceptive stimulus intensity was calibrated for each participant to be moderately painful (see below). At the end of the session, participants completed the NASA Task Load Index (NASA TLX; Hart & Staveland, 1988). Total session duration was 1.5 hr.

### 2.3 Cognitive inhibition abilities

Cognitive inhibition abilities were assessed using the following three paradigms:

The flanker task (Eriksen & Eriksen, 1974) was used to assess selective attention and interference control. Participants had to respond as fast as possible to the direction of a centrally presented arrow, while ignoring surrounding arrows (flankers). Incongruent flankers (pointing in the direction opposite to that of the central arrow) produce interference, resulting in a longer reaction time and more errors compared to congruent flankers, as participants need to exercise top‐down control (Diamond, 2013; Eriksen, 1995). In total, participants completed 20 randomized trials. The amount of interference was quantified by computing the difference score between the mean RT for incongruent and congruent trials, weighted by the percentage of correct responses (Mullane, Corkum, Klein, & McLaughlin, 2009). A lower score (i.e., a smaller difference between the incongruent and congruent condition) corresponds to a better selective attention ability. (Note that negative scores indicate a faster mean RT for incongruent compared to congruent trials).

A go/nogo task was used to measure prepotent response inhibition abilities (Cragg & Nation, 2008; Logan & Cowan, 1984). The ratio of *go* and *nogo* trials is usually skewed with more *go* than *nogo* trials, creating a prepotent response tendency which participants must then inhibit (Cragg & Nation, 2008). Participants were presented with a sequence of 20 circles, with the instruction to respond with a button press to plain green circles (*go* trials) and to withhold a response to a patterned circles (*nogo* trials). Performance on this task was quantified in terms of the average RT time, weighted by the percentage of correct responses. A lower score means better inhibition skills.

The colour‐word condition of the Stroop test (Stroop, 1935) was used to assess prepotent response inhibition abilities (Banich, 2009; Diamond, 2013; Friedman & Miyake, 2004). In the colour‐word condition of the test, participants typically have to name the colour of a colour word (such as “red”) that is written either in the same ink colour (congruent trial, e.g., the word “red” written in red ink) or another ink colour (incongruent trial, e.g., the word “red” written in blue ink). Participants completed 40 trials of a computerized version of the test, in which they had to indicate the colour in which a colour‐word (such as “red”) was presented, by pressing colour coded keys on a keyboard. Participants were instructed to read the word and not to apply any strategies (e.g., eye squinting). Accuracy and reaction times of responses were recorded. The amount of interference was assessed by computing the difference score between the mean RT for incongruent and congruent trials, weighted by the percentage of correct responses. Lower scores correspond to better interference control. (Note that negative scores indicate a faster mean RT for incongruent compared to congruent trials).

### 2.4 Questionnaires

The PCS assesses the tendency to engage in overly negative thought processes during anticipatory or actual pain (Sullivan et al., 1995). It is a 13‐item scale consisting of three subscales assessing rumination (4 items, e.g., “I keep thinking about how much it hurts”), magnification (3 items, e.g., “I become afraid that the pain will get worse”) and helplessness (6 items, e.g., “It's terrible and I think that it's never going to get any better”) in the face of pain. Participants rate each item on a 5‐point Likert scale, ranging from 0 = “not at all” to 4 = “all the time”. The total score ranges from 0 to 52, (rumination score range: 0–16; magnification score range: 0–12; helplessness score range: 0–24), with a higher score indicating a stronger tendency to catastrophize about pain. A validated German translation was published by Meyer, Sprott, and Mannion (2008).

The FPQ‐III is a 30‐item scale from McNeil and Rainwater (1998) and assesses pain‐related fear and anxiety on three subscales, namely, fear of minor pain (e.g., getting a paper‐cut on your finger), medical pain (e.g., receiving an injection in your arm), and severe pain (e.g., breaking your arm). Each subscale comprises 10 items. Participants rate each item on a 5‐point Likert scale, ranging from 1 = “not at all” to 5 = “extreme”. The scores for each subscale range from 10 to 50 and the total score ranges from 30 to 150, with higher scores indicating more fear of pain. A validated German translation exists (Kröner‐Herwig, Gaßmann, Tromsdorf, & Zahrend, 2012).

The NASA Task Load Index (TXL) is used to rate the subjective workload of a task on six dimensions, i.e., mental demand, physical demand, temporal demand, performance, effort and frustration level (Hart & Staveland, 1988). Each of these six items has to be rated on a 100 percent scale, ranging from “very low” to “very high”, which is divided into 20 intervals in increments of 5. Participants were instructed to complete the scale specifically in relation to the high load distraction task. A German translation was available from Pfendler (1991).

### 2.5 Pain stimuli—calibration procedure

Heat stimuli were administered to the volar surface of the left forearm using a contact thermal stimulator (25 × 50 mm; Somedic AB, Sweden). Prior to the pain distraction paradigm, nociceptive stimulus intensity was individually calibrated for each participant to account for interindividual differences in pain sensitivity. During the calibration, participants had to rate the intensity and unpleasantness of a pseudorandomized series of 10 heat stimuli (temperature range: 39–47°C) on visual analogue scales. The 200‐point intensity scale had three anchor points, namely “not warm” (0), “just pain” (100) and “unbearable pain” (200). The 100‐point unpleasantness scale ranged from “not unpleasant” (0) to “extremely unpleasant” (100); for more details, see Methods S1 in the Supplementary Materials. Heat stimuli lasted for a total of 16 s with a plateau phase of 10 s and ramp‐up/ramp‐down phases of 3 s each. For painful stimuli, the slope of the ramp‐up/down phases was 5°C/s and for non‐painful stimuli 3°C/s. Baseline temperature was set to 35°C. During stimulus presentation, a fixation cross was displayed in the centre of the screen followed by the presentation of the two scales after an interval of 4–8 s (average: 6 s). The next heat stimulus was presented 2–4 s (average: 3 s) after the rating of the previous stimulus.

The target temperature for the painful stimuli was determined by interpolating the resulting intensity ratings with the TREND function in MS Excel 2016 (Microsoft Excel) and identifying the temperature corresponding to a rating of 140 on the intensity VAS. This moderate pain level was selected based on pilot studies in our lab and ensured that the stimuli were clearly painful, yet avoided that they would be perceived as too intense to ignore them (see McCaul & Malott [1984] for a theoretical discussion of the role of noxious intensity levels in distraction from pain). Internal comparisons of *R* ^2^ values between linear and exponential functions showed that a linear function matched our calibration data the best. The target temperature for the non‐painful stimuli was set to 40°C for all participants.

### 2.6 Distraction paradigm

In the distraction paradigm, participants received 20 s trials of the n‐back working memory task in the high (2‐back) and low load (0‐back) condition, programmed in E‐Prime 2.0 (Psychology Software Tools, Pittsburgh, USA). In the 2‐back task, participants were presented with a sequence of letters (either C, F, J, N, Q, S, V or X) and had to indicate for each letter whether or not it was the same as the letter presented two steps back. The 0‐back task required participants to indicate whether the current letter was an “X” or not. Note that the 0‐back condition of the task differed from the 2‐back condition only in terms of the instructions.

In each trial, 25% of letters were targets. The first two letters of each trial were excluded from the analyses, as no comparison could be made with letters presented two steps back. In addition, the 2‐back task contained 12.5% lures (i.e., letters that were identical to the one presented one or three steps back) to increase task difficulty. In both tasks, no more than two target letters were shown in succession.

Participants first practiced both tasks, receiving auditory feedback for correct and incorrect responses. In this practice phase, we displayed the amount (total sum of all correct (+€0.05) and incorrect (−€0.05) responses) that participants would have earned or lost, at the end of each trial. This was implemented to increase motivation to perform well in both tasks (Van Damme, Legrain, Vogt, & Crombez, 2010; Verhoeven et al., 2010).

Using a procedure adapted from Buhle and Wager (2010), we continuously adjusted the difficulty of the 2‐back task to the individual's performance by adjusting the length of the inter‐character interval (i.e. task speed). Initial task speed for both tasks was derived from a prior on‐line calibration where participants completed 20 2‐back trials without performance feedback. In a staircase procedure, the interval duration (start: 2,000 ms) was increased or decreased after every two trials depending on the participants A′, a non‐parametric measure of performance (based on the participant's hit and false alarm rate; Stanislaw & Todorov, 1999). An A′ value of 1 means perfect performance whereas an A′ value of 0.5 means performance at chance (Stanislaw & Todorov, 1999). Our target level of performance was set to an A’ value of 0.85 in order to maintain a similar level of task difficulty across participants. If A′ was greater than 0.85 and the missing response rate was <25%, the inter‐character duration was reduced by the absolute value of 1,200* (A′−0.85). In case A′ was equal to or smaller than 0.85, the duration was increased by the absolute value of 1,200*(A′−0.85). The inter‐character interval was also increased if the missing response rate was equal or greater than 25%, even if A’ was equal or greater than 0.85 as missing responses had no effect on the false alarm rate and did thus not affect the A’ values. A constriction prevented the interval duration from decreasing beyond 100 ms. Note that we also considered responses with an RT <150 ms as incorrect as they are unlikely to reflect true responses (see Legrain, Crombez, Verhoeven, & Mouraux, 2011; Schmiedek, Li, & Lindenberger, 2009).

The distraction paradigm consisted of 32 trials in total; eight trials for each of the four conditions, namely warm/0‐back, warm/2‐back, pain/0‐back and pain/2‐back. The paradigm was divided into four blocks containing eight trials each (two for each condition), with a short break between blocks. Trial order was pseudorandom; no more than two painful stimuli or 2‐back tasks were presented in a row. Trial duration was always 20 s. Before each trial started, a cue word (“X‐target” for the 0‐back task, or “2‐back”) was presented for 5,000 ms, signalling the upcoming task. Each letter in a trial was presented for 500 ms, preceded by a fixation cross (250 ms) and followed by the adaptive inter‐character interval (min. 100 ms). During this interval, a blank screen was presented.

As a mnemonic for participants as to the current task instructions, all letters in the 0‐back task were presented in yellow and all letters in the 2‐back task in magenta (both on a black background). Four seconds after task onset, thermal stimulation started. Task speed was continuously adapted every two 2‐back trials (i.e., within and after every block), using the same procedure as during the initial calibration; the speed for the 0‐back task was always set to the same speed as the 2‐back task after each block.

The task trial and the thermal stimulation finished simultaneously, and after an interval of 4–8 s (average: 6 s) showing a fixation cross, participants were asked to rate the thermal stimulus on an intensity and unpleasantness VAS (unlimited time). After an inter‐trial interval of 4–8 s (average: 6 s) displaying a fixation cross, the next trial started.

### 2.7 Statistical analyses

Statistical analyses were performed using SPSS 25 (IBM SPSS Statistics). We assessed possible gender differences in the psychological and cognitive inhibition measures as well as VAS ratings and painful temperature in the distraction paradigm using independent sample *t*‐tests since some studies reported gender differences in pain catastrophizing and pain coping strategies (see e.g. D’Eon, Harris, & Ellis, 2004; Keogh & Eccleston, 2006; Sullivan, Tripp, & Santor, 2000).

To assess the magnitude of the distraction effect, intensity and unpleasantness ratings were both subjected to a repeated measures ANOVA with the within‐subject factors *temperature level* (warm vs. painful) and *task difficulty* (low load vs. high load). We also calculated the mean difference in intensity (∆I) and unpleasantness (∆U) ratings for painful stimuli for the two levels of *task difficulty* (low load/painful—high load/painful) as an index of the distraction effect size. We then correlated these distraction effect indices with the different psychological and cognitive measures using the non‐parametric Spearman correlation whenever the assumption of normality was violated as assessed with the Kolmogorov–Smirnov test, and a Pearson correlation if the assumption of normality was met. In addition, we tested significant correlations for interaction effects, using moderation analyses, as implemented in the PROCESS v3.3 macro in SPSS 25 (Hayes, 2017).

We used two‐tailed tests for all analyses unless we had a specific hypothesis with regard to the direction of the effect. The significance level was set to α = 0.05. In case of multiple comparisons, we used a Bonferroni‐corrected α. Partial eta squared (*n_p_* ^2^) effect size measures are reported for significant effects in the ANOVA models, where 0.01 represents a small effect, 0.06 represents a medium effect and 0.14 represents a large effect (Richardson, 2011).

## 3 RESULTS

### 3.1 Descriptive statistics

Two participants were removed from the initial sample of *N* = 41 participants, and statistical analyses were performed on the remaining 39 participants. The first participant was excluded because performance in the 2‐back task calibration was close to chance (average A’ below 0.60). The second one was excluded because intensity ratings for painful stimuli in the distraction paradigm were well below the pain threshold (*M* = 41.25; pain threshold = 100). Additionally, extreme outlier analyses revealed that three participants had an extremely large flanker effect (IQR: 145 ms) and five participants showed extremely long reaction times in the go/nogo task (IQR: 168 ms). As these outliers were most likely caused by a computer logging error, we excluded these participants from further statistical analyses involving these tasks.

A Kolmogorov–Smirnov test indicated that none of the cognitive measures were normally distributed (flanker: *D*[36] = 0.154, *p* = 0.031; go/nogo: *D*[34] = 0.209, *p* = 0.001; Stroop: *D*[39] = 0.154; *p* = 0.020) and neither were the intensity change scores (∆I), *D*[39] = 0.147, *p* = 0.033, nor the FPQ scores, *D*(39) = 0.142, *p* = 0.045. The unpleasantness change scores (∆U), *D*(39) = 0.134, *p* = 0.073, and the PCS scores, *D*(39) = 0.114, *p* = 0.200, were normally distributed. Also the assumption of normality conducted on the regression residuals for the moderation analysis was met, *D*(36) = 0.135, *p* = 0.095.

Descriptive statistics of the psychological and cognitive measures are given in Table 1. To assess possible gender differences in the psychometric characteristics of our sample, we subjected the total scores and subscale scores of the PCS and FPQ‐III as well as the flanker, go/nogo and Stroop outcome measures to independent sample *t*‐tests (2‐tailed). We observed no significant gender difference on any of these measures (all *t*s < 1.63, all *p*s > 0.111). Two‐tailed independent sample *t*‐tests also showed no difference between genders for painful temperature (on average: 46.60 ± 1.1°C) and intensity and unpleasantness ratings (all *t*s < 1.496, all *p*s > 0.143).

**TABLE 1:** Psychometric characteristics of the sample

Measure	Sample size (*N*)	Mean	*SD*	Sample scale range
*PCS total*	39	19.03	8.99	1–38
Rumination		7.64	3.85	0–14
Magnification		3.85	2.27	0–11
Helplessness		7.54	4.05	0–16
*FPQ‐III total*	39	79.10	16.25	39–111
Severe pain		32.56	7.24	16–46
Medical pain		25.85	6.34	11–35
Minor pain		20.69	6.03	10–35
*NASA TLX total*	39	65.85	13.43	39–90
Mental		13.82	3.67	4–20
Physical		5.54	4.08	1–16
Temporal		11.82	4.48	1–19
Performance		10.33	3.70	2–19
Effort		13.85	3.07	6–20
Frustration		10.49	4.73	2–19
*Flanker effect (ms)*	36^a^	87.25	111.27	−158 to 292^b^
RT (congruent)		557.64	106.10	403–876
RT (incongruent)		638.72	133.77	432–1,168
*Go/nogo effect (ms)*	34^a^	486.43	131.46	354–988
*Stroop effect (ms)*	39	230.41	254.54	−266 to 1,210^b^
RT (congruent)		961.13	215.35	685–1,629
RT (incongruent)		1,165.28	319.34	681–2,006

^a^ Extreme outliers (i.e., values greater or lower than 3x interquartile range; IQR: Q3‐Q1) were identified with boxplots and participants were subsequently removed from all analyses involving these tasks (flanker: 3; go/nogo: 5).

^b^ Flanker and Stroop interference scores were obtained by subtracting RT measures for congruent trials from incongruent trials, and weighting the resulting difference score by the percentage of correct responses.

### 3.2 Distraction effect

**FIGURE 1:**
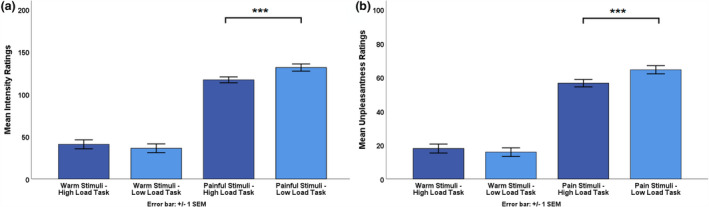
(a) Mean intensity ratings for the four different conditions. Bonferroni corrected paired sample *t*‐tests revealed that intensity ratings for painful, but not warm, stimuli differed significantly per task condition (distraction vs. control). (b) Mean unpleasantness ratings for the four different conditions. Bonferroni corrected paired sample *t*‐tests revealed that unpleasantness ratings for painful, but not warm, stimuli differed significantly per task condition (low vs. high load). Error bars reflect ± *SD*. Note that warm stimuli were significantly less intense and unpleasant than painful stimuli, irrespective of task difficulty, *p* < 0.001. For simplification, these differences are not marked as significant in the figure. ***p < 0.001

A repeated measures ANOVA with the within‐subject factors *temperature level* (warm vs. painful) and *task difficulty* (low load vs. high load) revealed a significant main effect for *temperature level*, *F*(1,38) = 261.54, *p* < 0.001, *n_p_* ^2^ = 0.87, on intensity ratings. Painful stimuli were perceived as significantly more intense than warm stimuli. Similarly, we found that painful stimuli were rated to be significantly more unpleasant than warm stimuli, *F*(1,38) = 288.82, *p* < 0.001, *n_p_* ^2^ = 0.88 (see Figure 1).

Results also revealed significant differences in intensity and unpleasantness ratings for *task difficulty*, *F*(1,38) = 7.66, *p* = 0.009, *n_p_^2^* = 0.168, and *F*(1,38) = 8.34, *p* = 0.006, *n_p_* ^2^ = 0.18, respectively.

Most importantly, we observed a significant interaction effect between *temperature level* (warm vs. painful) and *task difficulty* (low load vs. high load) for intensity ratings, *F*(1,38) = 36.87, *p* < 0.001., *n_p_^2^* = 0.49, and unpleasantness ratings, *F*(1,38) = 37.14, *p* < 0.001, *n_p_^2^* = 0.49. Bonferroni corrected paired sample *t*‐tests (critical *p* = 0.0125, *k* = 4) showed that intensity and unpleasantness ratings for painful stimuli were significantly lower when performing the high load compared to the low load task, *t*(38) = 5.37, *p* < 0.001 and *t*(38) = 5.05, *p* < 0.001, respectively (see Figure 1). Thus, in accordance with our hypothesis, results indicate that the more demanding high load task was indeed successful in distracting participants from pain.

### 3.3 Distraction effect and cognitive measures

To test whether cognitive inhibition abilities predict the magnitude of the distraction effect, we correlated performance on the flanker (*N* = 36), go/nogo (*N* = 34) and Stroop (*N* = 39) task with the intensity change score (∆I) and unpleasantness change score (∆U), using a one‐tailed Spearman correlation. We expected that better inhibition skills would lead to a larger distraction effect.

Results revealed a negative correlation between the flanker effect and ∆I, *r*_s_ = −0.279, *p* = 0.050 (*N* = 36), indicating that a smaller flanker effect, i.e., better selective attention abilities, were associated with a larger distraction effect size. However, we observed no relationship between ∆I and performance on the go/nogo (*r*_s_ = −0.132, *p* = 0.228; *N* = 34) or Stroop task (*r*_s_ = 0.015, *p* = 0.465; *N* = 39). Also, Spearman correlations for ∆U and cognitive inhibition abilities yielded no significant results (flanker: *r*_s_ = −0.187, *p* = 0.137; go/nogo: *r*_s_ = −0.122, *p* = 0.247; Stroop: *r*_s_ = −0.107, *p* = 0.258).

### 3.4 Distraction effect and psychological measures

A two‐tailed Spearman correlation revealed that ∆I correlated marginally with the PCS score (*N* = 39), *r*_s_ = 0.300, *p* = 0.064, but not with the fear of pain scor*e, p* = 0.102. We found no significant correlation between PCS and FPQ scores and ∆U (all *p*s > 0.08). (Also note that PCS and FPQ scores did not correlate with pain ratings averaged across the low load and high load condition; see Table S2 for the results.)

Given that pain catastrophizing seems to influence the distraction effect size, we ran a partial Spearman correlation (one‐tailed) for the distraction effect size and the flanker effect, with PCS scores as covariates. Controlling for PCS scores strengthened the correlation between flanker effect and ∆I (*r*_s_ = −0.318, *p* = 0.032), indicating that PCS scores may moderate the relationship between ∆I and cognitive inhibition/selective attention abilities.

To further explore this potential role of catastrophizing in the relationship between the distraction effect and the flanker effect, we ran a moderation analysis with PCS scores as moderator. To facilitate the interpretation of the coefficients, we standardized and mean‐centred PCS and flanker scores. Confidence intervals (95%) were computed using bootstrapping (5,000 samples).

The overall model accounted for a significant portion of variance of the magnitude of the distraction effect, *F*(3,32) = 4.79, *p* = 0.007, *R* ^2^ = 0.31. As can be seen in Table 2, better selective attention abilities were a significant predictor of ∆I (*p* = 0.024). PCS scores were marginally significant predictors of ∆I (*p* = 0.054). Moreover, the interaction between flanker task performance and PCS scores was significant in predicting ∆I (*p* = 0.030).

**TABLE 2:** Moderation analysis

	Beta coefficients	*SE*	*t*	*p*	LLCI (95%)	ULCI (95%)
Constant	16.37	2.55	6.43	<0.001	11.1783	21.5535
Flanker	−6.18	2.61	−2.37	0.024	−11.4901	−0.8670
PCS total	5.27	2.63	2.00	0.054	−0.0866	10.6280
Flanker × PCS total	−7.22	3.17	−2.28	0.030	−13.6849	−0.7578

**FIGURE 2:**
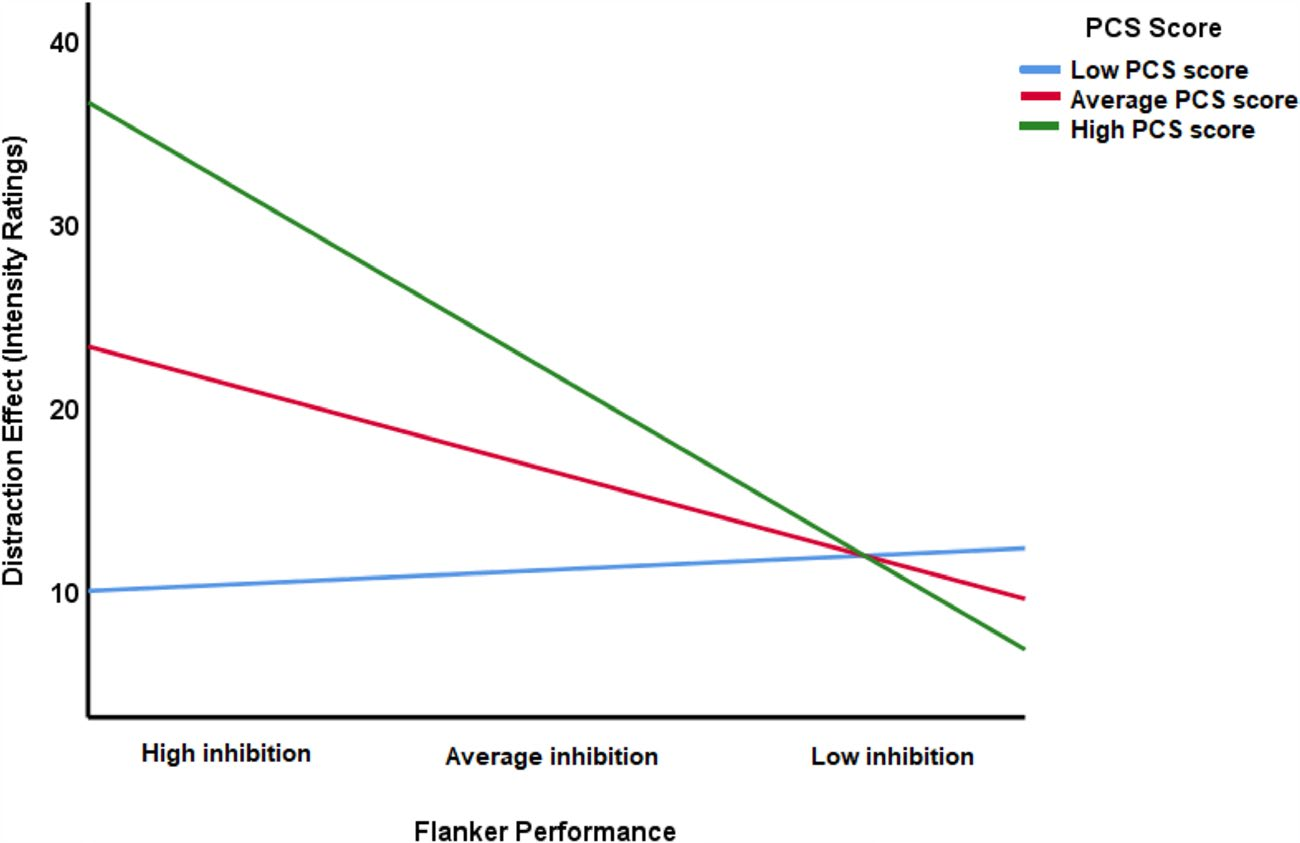
Pain catastrophizing moderated the relationship between the flanker effect (a smaller effect is assumed to reflect better selective attention abilities) and the size of the distraction effect on the intensity rating scale. Better selective attention abilities were associated with a significantly larger distraction effect, but only if participants scored high on the PCS. Note that the here depicted division of PCS scores into low, medium and high is just for illustration purposes, and that PCS scores were treated as a continuous variable in the moderation analysis

Probing the interaction with conditional effect analyses revealed that better performance on the flanker task had no effect on the magnitude of the distraction effect for participants scoring low on the PCS (1 *SD* below the mean), *b* = 1.04, *t*(32) = 0.28, *p* = 0.782. However, for participants with average (mean) or high PCS scores (1 *SD* above the mean), better performance on the flanker task was associated with a larger distraction effect, *b* = −6.18, *t*(32) = −2.37, *p* = 0.024, and *b* = −13.40, *t*(32) = −3.02, *p* = 0.005, respectively (see Figure 2). This suggests a positive relationship between selective attention abilities and the efficacy of task‐induced analgesia for average to high pain catastrophizers. Exploratory analyses of the PCS subscalses (rumination, magnification, helplessness) showed that no specific subscale was driving the moderation effect (see Table S1 for these results). Rather, the *R* ^2^ values suggest that the PCS total score accounted for the highest variance in the dependent variable.

## 4 DISCUSSION

We investigated the influence of cognitive inhibition abilities and negative pain‐related cognitions, i.e., pain catastrophizing and fear of pain, on distraction from pain in healthy young adults. As expected, a working memory task with high cognitive load significantly reduced perceived intensity and unpleasantness of concurrent nociceptive stimuli, compared to the same task with low cognitive load. Interestingly, we found greater task‐related analgesia in individuals with better cognitive inhibition abilities; participants who had better selective attention skills (as measured by the flanker task) tended to experience more pain reduction. In addition, the effectiveness of distraction tended to be larger in participants who were high in pain catastrophizing. A moderation analysis targeting the roles of pain catastrophizing and the flanker effect in predicting the distraction effect size revealed a significant interaction: Participants who performed well on the flanker task had a larger distraction effect, but only when they were average to high pain catastrophizers.

While the interruptive effect of pain on executive functions has been subject of numerous studies in chronic pain patients (Berryman et al., 2014) as well as healthy individuals (see e.g. Attridge, Noonan, Eccleston, & Keogh, 2015; Buhle & Wager, 2010; Crombez, Eccleston, Baeyens, & Eelen, 1996; Moore, Keogh, & Eccleston, 2012), our study is one of the first to find that executive functions, specifically selective attention, may be directly involved in the attentional modulation of pain and predict the efficacy of a distractive task. Better selective attention abilities probably help participants to sustain their attention to the task and to ignore the nociceptive stimulus. The lack of significant findings in previous research on the role of executive functions (Verhoeven et al., 2011, 2014) may have different reasons. Most studies, for instance, relied on tonic pain stimulation, such as the cold pressor test, with the disadvantage that the pain intensity may differ from individual to individual and fluctuate over time (Verhoeven et al., 2011, 2012; Wohlheiter & Dahlquist, 2012; Zhou et al., 2015). Moreover, in contrast with most other studies, we calibrated the distraction task difficulty for each individual. This may be important as research shows that different levels of cognitive load and pain intensity levels may result in complex interactions (Moore, Eccleston, & Keogh, 2017; Romero, Straube, Nitsch, Miltner, & Weiss, 2013; Seminowicz & Davis, 2006).

Our results suggest that the association between the distraction effect size and inhibition abilities might be quite specific to the construct measured with the flanker task. Although the degree to which the flanker task and the Stroop and go/nogo task measure the same construct is still an open question (see Bender, Filmer, Garner, Naughtin, & Dux, 2016; Friedman & Miyake, 2004), only the flanker task has been associated with focused attention/selective enhancement for target stimuli, i.e., resistance to distractor interference. In contrast, the go/nogo and Stroop task are assumed to measure the ability to override prepotent/habitual responses (see Friedman & Miyake, 2004). In our paradigm, the ability to resist distractor (i.e., pain) interference and to direct attentional resources to the distractive task may have been more relevant than the ability to inhibit prepotent responses. This is in line with other pain distraction studies that found no influence of prepotent inhibition abilities (measured with a modified version of the anti‐saccade task or the Stroop colour‐word test) on task‐induced analgesia (Verhoeven et al., 2011, 2014). However, it is noteworthy that prepotent response inhibition abilities have been shown to exert a protective effect against pain‐induced interference on task performance, with faster reaction times and smaller response variation (Karsdorp et al., 2014; Verhoeven et al., 2011). Our study design did not allow us to assess pain‐induced task interference (due to the continuous adaptation of the distraction task difficulty), but this would be interesting to confirm in future studies.

To our knowledge, the only other study that has directly investigated the effects of selective attention on distraction from pain, did this in children aged 3–6, using the Visual Attention subtest of the Developmental Neuropsychological Assessment (Wohlheiter & Dahlquist, 2012). Results revealed that children with a better selective attention ability had a higher pain tolerance, but selective attention skills did not modulate the increase in pain tolerance due to a distractive video game (Wohlheiter & Dahlquist, 2012). This contrasting finding could be explained by differences in the development of the attentional network in children and adults (see Rueda et al., 2004).

Interestingly, distraction effect size not only depended on selective attention abilities but also on the level of pain catastrophizing. The finding that distraction was most effective in high pain catastrophizers is consistent with a study on patients with persistent spinal pain, in which the effects of a sustained handgrip task on the temporal summation of mechanical pain (TSP) were assessed (Schreiber et al., 2014). In this study, high pain catastrophizers showed an amplified TSP without distraction, but reported a similar level of TSP in the presence of a distractive cognitive mechanical task that required participants to maintain a specific handgrip strength on a dynamometer. Other studies, however, found distraction from pain to be less effective in high compared to low catastrophizers (Heyneman et al., 1990; Prins et al., 2014; Verhoeven et al., 2012). Several methodological differences between these studies may explain these apparently conflicting findings.

First of all, research suggests that high pain catastrophizers may be particularly responsive to task characteristics, such as motivational incentives. Increasing the motivational relevance of the distraction task by providing a monetary incentive neutralized the detrimental effects of pain catastrophizing on the distraction effect size (Verhoeven et al., 2010). Consistent with this notion, Van Damme, Crombez, and Eccleston (2003) reported that high pain catastrophizers not only had difficulties to disengage from pain cues in a cueing paradigm, but also showed a stronger attentional engagement to the neutral cue (a tone) compared to low pain catastrophizers, presumably because of its safety value. This suggests that high pain catastrophizers may have a high intrinsic motivation to direct their attention to non‐threatening cues, despite difficulties to disengage from pain cues. Consequently, high catastrophizers may benefit equally well as, or even more than, low catastrophizers from the analgesic effects of a distractive task, provided that the distractive task surpasses the painful sensation in terms of motivational relevance. The task characteristics of the present study may have been particularly suited to engage the attention of high pain catastrophizers as we offered a monetary incentive for good task performance and used a demanding working‐memory task as distractor task that continuously required attentional resources (Buhle & Wager, 2010). In addition, thermal stimulation started only 4 s after task onset, which may have helped participants to direct and sustain their attention to the task from the beginning (Buhle & Wager, 2010; Zhou et al., 2015). Moreover, as painful stimuli were calibrated to be only moderately painful, participants high in pain catastrophizing may have found it easier to disengage from them as compared to stimuli of higher pain intensity (Seminowicz & Davis, 2006).

It should be noted that only pain catastrophizing, but not fear of pain, moderated the relationship between the distraction effect and selective attention abilities. Neither the PCS nor the FPQ‐III predicted pain ratings as such (see Table S2), presumably because of the modulation of our pain stimuli by the distraction paradigm. A potential explanation as to why the PCS better predicted the size of the distraction effect could be that the PCS assesses the cognitive‐affective response to actual or anticipated pain, including one's beliefs about the ability to inhibit pain‐related thoughts (Quartana, Campbell, & Edwards, 2009), whereas the FPQ‐III assesses primarily fear responses to specific pain situations (McNeil & Rainwater, 1998).

The present study has some limitations. First, participants were young and healthy university students with overall good inhibition abilities, which limits the generalizability of the results to other populations. In addition to this, inhibition abilities were assessed with a web‐based application that did not allow us to retrieve individual reaction times for each trial and thus limited the choice of performance measures. Moreover, computerized versions of the Stroop test have been found to lead to less interference than the conventional pen‐and‐paper version and may even measure a different construct (Penner et al., 2012). Future research could address these issues by using a wider range of cognitive inhibition and attention tests, such as the attentional network task (Fan, McCandliss, Sommer, Raz, & Posner, 2002), and by investigating the relationship between cognitive inhibition abilities and the effectiveness of distraction in populations with impaired or reduced executive functions, such as chronic pain patients, patients with frontal lobe damage or older individuals (Van Hooren et al., 2007).

In conclusion, our results support the notion that selective attention is an important factor underlying the effectiveness of task‐related analgesia, presumably by enabling one to sustain attention towards the distractive task, while resisting the reflex to focus on pain. However, our results also suggest that it is crucial to assess individual differences in negative pain‐related cognitions, such as pain catastrophizing, as they may interact with the influence of selective attention abilities.

## CONFLICTS OF INTEREST

All authors declare that no conflicts of interest exist.

## AUTHOR CONTRIBUTIONS

The study was designed by MVDM and implemented by KMR. SG was involved in the data collection. KMR analysed the data and wrote the first draft of the manuscript. All authors contributed critically to the discussion and interpretation of the results and commented on the manuscript.

## Supporting information

Methods S1

Table S1

Table S2
