# Supplementary material for: Distraction from pain: The role of selective attention and pain catastrophizing
Source: Eur J Pain. 2020 Aug 13;24(10):1880–91. doi: 10.1002/ejp.1634 (PMC7689692; doi:10.1002/ejp.1634)
Supplement: Supplementary file 1 — Methods S1 [file EJP-24-1880-s001.docx]

**methodsS1: Specification of rating scales**

Participants rated the intensity and unpleasantness of the thermal stimuli on two scales. They first rated the intensity of a stimulus on a 200-point scale with three anchor points (0 = “not warm”, 100 = “just pain”, 200 = “unbearable pain”). Following this, they rated the unpleasantness of the stimulus on a 100-point scale with two anchor points (0 = “not unpleasant”, 100 = “extremely unpleasant”).

Two different scale ranges for intensity and unpleasantness ratings were used as participants were not expected to rate stimuli as pleasant. (Participants could give a rating of 0 to indicate if a stimulus was not perceived as unpleasant). Therefore, this scale only comprised an “unpleasantness” dimension, as opposed to the intensity scale which was divided into a “non-painful” and a “painful” dimension. The unpleasantness scale thus required a less fine-grained resolution. Note that our participants were unaware of how many points each scale had, and rated the stimuli with reference only to the anchor point labels.
