# Supplementary material for: Distraction from pain: The role of selective attention and pain catastrophizing
Source: Eur J Pain. 2020 Aug 13;24(10):1880–91. doi: 10.1002/ejp.1634 (PMC7689692; doi:10.1002/ejp.1634)
Supplement: Supplementary file 2 — Table S1 [file EJP-24-1880-s002.docx]

**Table S1. Moderation analyses of PCS subscales**

*PCS rumination subscale:*

The overall model accounted for a significant portion of variance of the magnitude of the distraction effect, *F*(3,32) = 4.39, *p* = .011, *R*^2^ = .29.

a) Moderation analysis for the PCS rumination subscale.

|  | Beta coefficients | SE | *t* | *p* | LLCI (95%) | ULCI (95%) |
| --- | --- | --- | --- | --- | --- | --- |
| Constant | 15.87 | 2.55 | 6.22 | < .001 | 10.67 | 21.06 |
| Flanker | -4.83 | 2.59 | -1.87 | .071 | -10.10 | .44 |
| PCS Rumination | 5.00 | 2.73 | 1.83 | .076 | -.55 | 10.55 |
| Flanker x PCS Rumination | -5.34 | 2.71 | -1.97 | .057 | -10.86 | .18 |

*PCS magnification subscale:*

The overall model did not account for a significant portion of variance of the magnitude of the distraction effect, *F*(3,32) = 2.76, *p* = .059, *R*^2^ = .21.

b) Moderation analysis for the PCS magnification subscale.

|  | Beta coefficients | SE | *t* | *p* | LLCI (95%) | ULCI (95%) |
| --- | --- | --- | --- | --- | --- | --- |
| Constant | 15.31 | 2.69 | 5.70 | < .001 | 9.85 | 20.78 |
| Flanker | -4.34 | 2.73 | -1.59 | .122 | -9.89 | 1.22 |
| PCS Magnification | 5.62 | 2.72 | 2.06 | .047 | .07 | 11.17 |
| Flanker x PCS Magnification | -4.98 | 3.63 | -1.37 | .180 | -12.36 | 2.41 |

*PCS helplessness subscale:*

The overall model accounted for a significant portion of variance of the magnitude of the distraction effect, *F*(3,32) = 4.02, *p* = .016, *R*^2^ = .27.

c) Moderation analysis for the PCS helplessness subscale.

|  | Beta coefficients | SE | *t* | *p* | LLCI (95%) | ULCI (95%) |
| --- | --- | --- | --- | --- | --- | --- |
| Constant | 17.16 | 2.68 | 6.40 | < .001 | 11.69 | 22.62 |
| Flanker | -8.10 | 2.91 | -2.78 | .009 | -14.03 | -2.17 |
| PCS Helplessness | 5.03 | 2.68 | 1.88 | .070 | -.43 | 10.48 |
| Flanker x PCS Helplessness | -8.27 | 3.46 | -2.39 | .023 | -15.32 | -1.22 |
