# Supplementary material for: Distraction from pain: The role of selective attention and pain catastrophizing
Source: Eur J Pain. 2020 Aug 13;24(10):1880–91. doi: 10.1002/ejp.1634 (PMC7689692; doi:10.1002/ejp.1634)
Supplement: Supplementary file 3 — Table S2 [file EJP-24-1880-s003.docx]

**Table S2. Pearson and Spearman correlations for the PCS and FPQ-III and pain ratings.**

a) Pearson correlation ^a^ coefficients for the PCS and pain ratings (N = 39).

|  | Pain intensity | Pain unpleasantness |
| --- | --- | --- |
| PCS total | .141 | .032 |
| PCS rumination | .112 | .048 |
| PCS magnification | .086 | -.086 |
| PCS helplessness | .158 | .074 |

b) Spearman correlation ^a^ coefficients for the FPQ-III and pain ratings (N = 39).

|  | Pain intensity | Pain unpleasantness |
| --- | --- | --- |
| FPQ-III total | .235 | .181 |
| FPQ-III minor pain | .276 | .211 |
| FPQ-III medical pain | .169 | .157 |
| FPQ-III severe pain | .168 | .127 |

^a^ A Kolmogorov-Smirnov test indicated that the total PCS scores and pain ratings were normally distributed (PCS: D(39) = .114, *p* = .200; pain intensity rating: D(39) = .075; *p* = .200; pain unpleasantness ratings: D(39) = .102; p = .200), but not the total FPQ-III scores, D(39) = .142, *p* = .045. The tables below show the Pearson correlation coefficients for the PCS and pain ratings (averaged over ratings in the low and high load condition) and the Spearman correlation coefficients for the FPQ-III and pain ratings (*N* = 39). None of the correlations were significant (all p>.05).
